# Welfare Concerns for Mounted Load Carrying by Working Donkeys in Pakistan

**DOI:** 10.3389/fvets.2022.886020

**Published:** 2022-05-27

**Authors:** Syed S. U. H. Bukhari, Sarah M. Rosanowski, Alan G. McElligott, Rebecca S. V. Parkes

**Affiliations:** ^1^Department of Veterinary Clinical Sciences, Jockey Club College of Veterinary Medicine and Life Sciences, City University of Hong Kong, Hong Kong, Hong Kong SAR, China; ^2^Equine Veterinary Consultants Limited, Hong Kong, Hong Kong SAR, China; ^3^Department of Infectious Diseases and Public Health, Jockey Club College of Veterinary Medicine and Life Sciences, City University of Hong Kong, Hong Kong, Hong Kong SAR, China; ^4^Centre for Animal Health and Welfare, Jockey Club College of Veterinary Medicine and Life Sciences, City University of Hong Kong, Hong Kong, Hong Kong SAR, China

**Keywords:** animal welfare, donkey welfare, loading practices, overloading of donkeys, working equids

## Abstract

Working donkeys (*Equus asinus*) are vital to people's livelihoods. They are essential for carrying goods, however, globally, overloading is one of the primary welfare concerns for working donkeys. We studied mounted load carrying by donkeys and associated factors in Pakistan. A cross-sectional study of donkey owners (*n* = 332) was conducted, and interviews were undertaken based on a questionnaire. Owners estimated that the median weight of their donkeys was 110 kg [interquartile range (IQR) 100–120 kg], and that they carried a median mounted load of 81.5 kg (IQR 63–99 kg). We found that 87.4% of donkeys carried a load above 50% of their bodyweight ratio (BWR), the median BWR carried was 77.1% (IQR 54.5–90.7%), and 25.3% of donkeys carried above 90% BWR. Donkeys that were loaded at more than 50% BWR were more likely to adopt sternal recumbency compared to donkeys loaded with less weight (*P* = 0.01). Donkeys carrying construction material were more likely to carry more than the median BWR, when compared to domestic loads (*P* < 0.001). Younger donkeys aged between one and 5 years carried more than the median BWR compared to those aged over 15 years (*P* = 0.03). For the models with donkeys carrying median BWR and above 90% BWR, those working in peri-urban and urban areas were more likely to carry a greater BWR than donkeys working in rural areas (*P* < 0.001; *P* < 0.001, respectively). For donkeys carrying more than 90% BWR, mixed breed donkeys carried higher loads compared to other breeds of donkeys (*P* < 0.001). Overloading based on current recommendations (50% BWR) was common, with the majority (87.4%) of donkeys reported to carry more than the recommended 50% limit. This survey provides evidence of on-the-ground working practices and factors associated with mounted load carrying, which is critical for developing evidence-based recommendations for loading, in order to improve the welfare of working donkeys.

## Introduction

Donkeys have played an essential role in developing human civilizations (1). There are approximately 50.5 million donkeys globally (2), benefiting around 600 million people and playing a vital role in the livelihood of poor and vulnerable communities in lower-middle income countries (LMICs) (1, 3–7). The importance of working donkeys for their owner's livelihoods and the economies of developing countries is well known (3, 5, 7); for example, in Senegal, draft donkeys contribute 74% of their driver's annual income (8). However, research has not yet quantified the value of working donkeys to the overall economies of LMICs (7). This may be why their importance has often been overlooked in government-level animal welfare policies (9). As such little is done to safeguard donkey welfare (10), leading to compromised welfare due to harsh working conditions, lack of legislation, and marginalization of both donkeys and donkey owning communities (3, 7).

**Figure 1:**
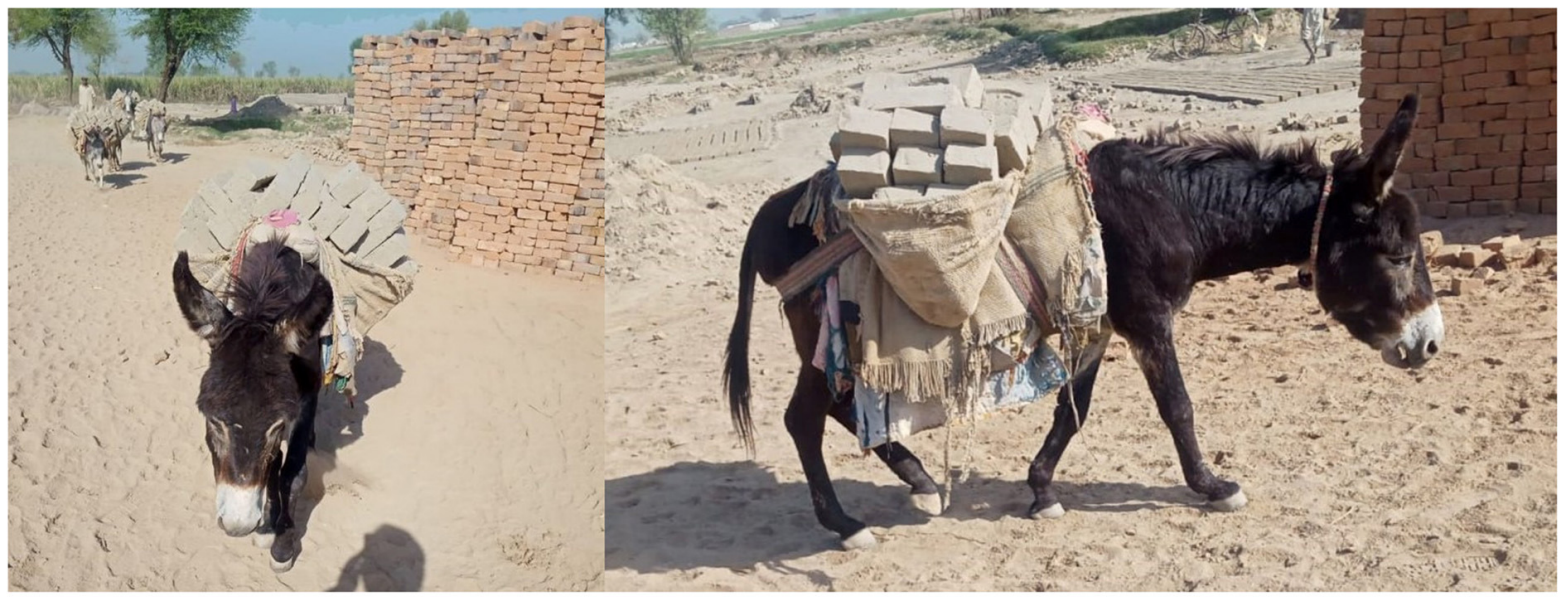
Mounted load carrying by donkeys in a brick kiln production system in Pakistan. Photo: Syed S. U. H. Bukhari.

Donkeys are used in a variety of settings, across rural, peri-urban, and urban areas (10) for plowing, fallowing, cultivation, and human transportation (3, 11). Donkeys are also used as pack animals for the transportation of construction, agricultural products, and domestic loads (3, 5, 7), including brick production (Figure 1). One of the most severe problems working donkeys experience is overwork and overloading (12–14). Overloading can be defined as the amount of weight that disrupts gait rhythm, resulting in lameness and behavioral changes (7). Some of the most common welfare issues documented in load carrying donkeys are skin sores and lesions, poor physical condition, chronic back pain, exhaustion, wounds, fractures, heat stress, dehydration, sprains, lameness, colic, metabolic disorders, myopathies, fear of humans, infrequent feeding, and hypoglycemia (11, 15, 16). Donkeys carry tons of mounted weight every day, which likely exceeds their natural weight carrying ability (7), and they work for extended periods of time (3, 7, 17). It has been reported that donkeys work for up to 12 h a day in Ethiopia, and cover a distance of more than 30 km a day in Morocco (17, 18). The working schedule of donkeys in Pakistan is currently not documented.

Animal welfare can be defined as the state of the animal's body and mind, and the extent to which its nature is satisfied (19). High workload and unsafe practices can contribute to poor working donkey welfare (3, 7), as overloading has been identified as impacting on equid behavioral, biochemical, biomechanical, and physiological characteristics (7). Behaviors associated with heavy loads include a reluctance or refusal to move forward and falling down, constant movement of the head with ears back, aggression, defecation, ear lifting, tail swishing, sniffing and moving backward, heavy and rapid panting, and reduced responsiveness (7, 10, 15). Excessive mounted load causes a number of internal biochemical changes, for example, a rise in blood lactate, nitrates, nitrites, and cortisol concentration (7, 15). Overloading can also affect gait biomechanics in horses, as it disturbs stride parameters, gait stability and symmetry. It is not known yet if these effects are evident in donkeys but it is highly likely (7). Finally, loading induces changes in multiple physiological indicators, for example, increase in heart rate, respiration rate, rectal temperature, and hematocrit. Mounted load-associated work also induces changes in muscle composition. However, heart rate variability decreases with heavier mounted load compared to lighter weights (7, 16).

There is little research regarding mounted load-carrying limitations of working donkeys. The maximum load recommended for a fit donkey in the UK, with a well-balanced load, is 50 kg (20), which is approximately 28% of an adult donkey's bodyweight. This 50 kg recommendation is not evidence-based, and refers to donkeys in the UK, which typically are in good body condition and are larger (21) than working donkeys in LMICs (22). Current guidelines for working donkeys based on research carried out in India suggest that donkeys can safely carry loads of up to 50% bodyweight (23). Donkeys in some LMICs have been reported to carry as much as 75% of their bodyweight (24). However, there is evidence of donkeys carrying up to 117% of their body weight in Pakistan (7, 25). Even conservative estimates indicate that these Pakistani donkeys carry more than their own bodyweight, which is one of the causes of compromised welfare (7). An increase in weight of the mounted load causes an increase in prevalence of skin wounds in working donkeys (17). Previous research has identified more hoof and gait abnormalities, tendon issues, joint swelling and soft tissue injuries, in older compared to younger donkeys. Older donkeys also had lower body condition scores (26). The current study aimed quantify demographics of donkeys, donkey loading practices, and factors related to mounted load carrying, in order to identify factors that may impact donkey welfare in Pakistan.

## Materials and Methods

### Study Area and Study Design

**Figure 2:**
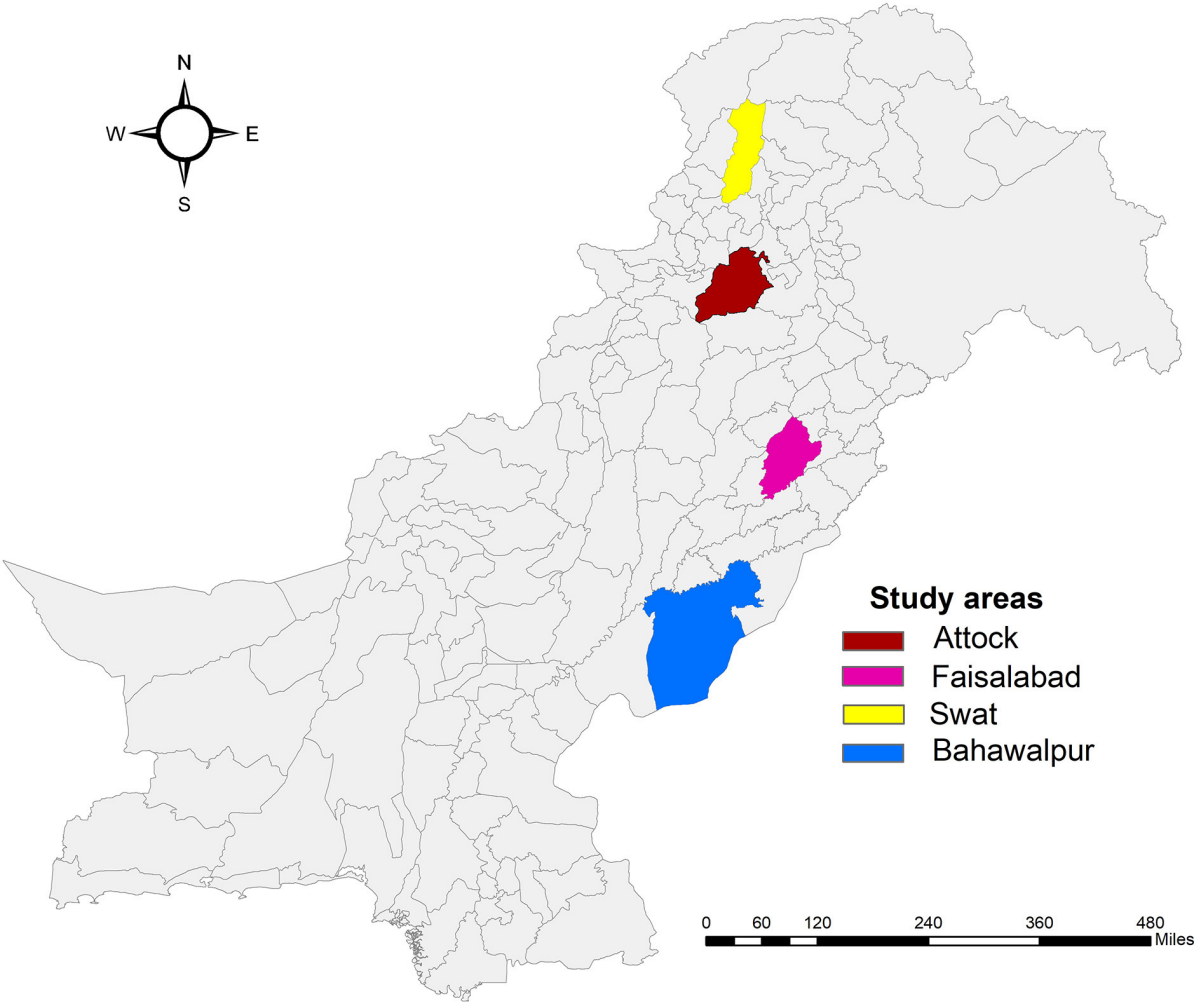
A map of Pakistan showing the locations of the four regions in which the study was conducted.

We carried out a cross-sectional survey of donkey owners in four of Pakistan's regions (Swat, Attock, Faisalabad, and Bahawalpur; Figure 2). These regions were selected based on their topography and varying climatic conditions: mountainous, arid, irrigated plains, and sandy desert, respectively (27). Different topographic regions were selected because working equids face different challenges in different communities and geographic sites (9, 28). The four regions cover almost 39,815 km^2^ (almost 4.5% of the country) of Pakistan. Swat (34°45' latitude, 72°54' longitude) is a mountainous region with an elevation of 2,591 m above sea level. The maximum average monthly temperature (37°C) remains during July, and the minimum average monthly temperature (0°C) is recorded during January. In Swat, annual rainfall ranges between 1,200–1,400 mm (27). Attock (32°55' latitude, 72°51' longitude) is an arid and semi-hilly region with an elevation of 519 m above sea level. The maximum average monthly temperature (38°C) occurs in June, with the minimum average monthly temperature (3°C) recorded in January. In Attock, annual rainfalls range between 900–1,000 mm (27). Faisalabad (31°26' latitude, 73°08' longitude) is among the irrigated plains of Pakistan, with an elevation of 185 m above sea level. The maximum average monthly temperature (41°C) occurs in June, with the minimum average monthly temperature (5°C) in January. In Faisalabad, annual rainfalls range between 300–400 mm (27). Most of Bahawalpur (28°39' latitude, 70°41' longitude) is a sandy desert region with an elevation of 88m above sea level. The maximum average monthly temperature (42°C) occurs in June, with the minimum average monthly temperature (4°C) in January. In this region, annual rainfalls range between 100–150 mm (27).

### Questionnaire Design

A questionnaire was developed to assess demographic characteristics, practices, and factors associated with mounted load carrying. The questions were designed based on field conversations with donkey owners combined with recent field experience of registered equine veterinarians in the four selected study regions. The questionnaire consisted of both open-ended and closed questions. The first section of the questionnaire consisted of informed verbal consent of donkey owners. The second section included information regarding the demographics of the owner, and the signalment of the donkey. The third section contained questions on the loading practices. In this section we asked about the weight of the donkey, weight of mounted load, whether the donkey adopted sternal recumbency when loaded, type of saddle, type of load, distance traveled, working terrain, working speed, daily working hours, signs of lameness, and daily income. The bodyweight of donkeys and the weight of any mounted loads as a part of their regular loading practices were estimated by the donkey owners. In some cases, donkey owners are able to weigh their donkeys on scales at nearby dairy farms or had recently weighed their donkey. Other owners estimated their donkey's weight. The weight of the mounted load was estimated depending on the items carried. For bricks, mounted load weight estimation was done by multiplying the number of bricks by the known weight of one brick. The weight of commercially packaged items was written on packaging, for example, a bag of cement, a bag of wheat grain, bags of fertilizers etc. The weight of liquids such as milk, oil, and water containers were estimated by number of liters in one can and the number of cans carried. Donkey owners were asked about sternal recumbency—whether the owner had previously noted a donkey trying to adopt sternal recumbency after loading—and lameness. The sternal recumbency and lameness could be observed by the owner without knowing the cause and to ensure clarity, the definition of sternal recumbency and lameness were explained if needed. Sternal recumbency was chosen as anecdotally, donkeys have been reported to perform this behavior under load.

A pilot study was conducted to optimize questions being asked, address discrepancies and check how much time the questionnaire took to complete. Information was collected from 24 randomly selected donkey owners, six from each of the four target regions (29). The time to complete the survey was 8–10 min. Surveys were all conducted verbally due to low literacy rates in the surveyed population. None of the data gathered from the pilot survey were included in the final analysis.

### Data Collection

The survey was conducted by equine veterinarians. They verbally explained the study, its purpose, and its methods. The donkey owners were approached based on convenience sampling and willingness to participate. Donkey owners were identified for inclusion in the study at the work location and on the basis of interviewer knowledge of the owners at a local level. Then the age of the owner was determined verbally and if they were more than 18 years old, they were recruited for the interview. Their informed verbal consent was taken before the start of the interview. Once donkey owners had provided consent to participate, interviews were undertaken based on the pre-designed questionnaire. The number of donkeys per owner varied. If an owner had more than one donkey, he answered the questions for one donkey. A total of 332 donkey owners participated. They had the opportunity to ask questions, and all their questions were answered appropriately. The interviewer signed a “participant informed verbal consent form”. A third person signed the witness statement (witness, to ensure appropriate exchange of information) on “participant informed verbal consent form” according to existing survey guidelines (30, 31). Face-to-face interviews were conducted to collect the required data, based on the pre-structured questionnaire which was in English. However, interviews were delivered in the local languages (Urdu, Pashtu, Hindko, Pothwari, Punjabi, Saraiki) after translation by the interviewers who were equine veterinarians and fluent in both English and the respective local languages. This approach was used to maximize the accuracy of responses and minimize any confusion concerning the scientific terminology used according to existing survey guidelines (30, 31). The questionnaire can be found in Supplementary Table 1.

### Statistical Analysis

The continuous data (weight of donkey, weight of the load, daily income generated by the donkey, and distance traveled per day) were presented in the form of median, interquartile range (IQR), minimum, and maximum. All the categorical data were described as frequency and percentage.

#### Outcome Variables

The following formula was used to calculate the percent bodyweight ratios (%BWR) for all donkeys,


(1) $$\%BWR\;=\;\frac{\text{Weight}\;\text{of}\;\text{Mounted}\;\text{Load}}{\text{Weight}\;\text{of}\;\text{Donkey}}\times 100\;$$

Three new binary outcome variables were created and labeled (1) 50% BWR, (2) median %BWR and (3) high %BWR. The first binary outcome variable had a cut-off BWR of 50% and was selected as an outcome variable based on existing guidelines which suggest that a donkey can safely carry up to 50% of their bodyweight (23). The second binary outcome variable had a cut-off BWR was the median of percent BWR in the population investigated, with half of the donkeys carrying above the median %BWR. The third binary outcome variable had a cut-off BWR of 90% and represented the upper quartile of our study population.

#### Exposure Variables

The variables considered in the logistic regression models were area (urban, peri-urban and rural), donkey age, donkey sex, breed (Sperki, Shinghari, Indian and mixed breed), type of saddle used, working terrain (mixed, plains, steep) and speed (walking or trotting). Continuous variables were non-normally distributed and were included in the model as categorical variables based on quartiles. Continuous variables were distance covered per day (in km) and earnings of a donkey in Pakistan rupees (PKR). Two donkey behaviors, sternal recumbency when loaded (yes/no) and lameness signs while working (yes/no) were included. Donkey breed and age were further categorized as binary variables, i.e., mixed breed and other breeds during multivariable modeling.

#### Univariable and Multivariable Regression Models

Univariable and multivariable logistic regression models were used to determine explanatory variables associated with mounted loads. Three multivariable logistic regression models were developed to investigate factors associated with each of the outcome variables—high %BWR, median %BWR, and 50% BWR. Exposure variables were screened using univariable logistic regression model for each outcome variable. Univariable regression models are provided in Supplementary Table 2. Exposure variables with a likelihood ratio test (LRT) *P-*value <0.20 were selected for inclusion in the multivariable model for that outcome. A preliminary multivariable model was built using a manual backward stepwise method of elimination in which variables were retained in the final multivariable regression model if the LRT *P-*value was <0.05. The LRT was used as the primary selection criterion. Confounding was assessed throughout the multivariable model building, with variables changing the odds ratio (OR) more than 10% retained in the final model. The goodness-of-fit of the logistic regression models was assessed using the Hosmer-Lemeshow test. All statistical analyses were conducted using Stata IC version 17 (32).

### Ethical Approval

This study was approved by the Human Subjects Ethics Sub-Committee, City University of Hong Kong (Approval reference no. JCC2021AY003).

## Results

### Demographic Characteristics

In total, 332 donkey owners agreed to participate. The demographics of the owners and donkey signalment are presented in Table 1. The majority of questionnaire participants (98.5%; *n* = 327) were men. Both male (54.5%; *n* = 181) and female (45.2%; *n* = 150) donkeys were used for load-carrying work. The majority of donkeys (58.1%; *n* = 193) were aged between 6 to 10 years. Donkeys worked in rural (48.7%; *n* = 162), peri-urban (38.3%; *n* = 127), and urban (13.0%; *n* = 43) areas. The distance covered by donkeys during their working day was a median of 8 km (IQR 3–17 km). Daily earnings were a median of 685 PKR (IQR 450–900) (USD$3.87 (IQR $2.54–$5.08)).

**Table 1:** Demographic characteristics of the donkey owners and donkeys.

**Variable**	**Category**	**Number**	**Percentage (%)**
Owner age (years)	<31	75	22.6
	31–40	141	42.5
	41–50	97	29.2
	>50	19	5.7
Owner gender	Male	327	98.5
	Female	5	1.5
Area	Rural	162	48.8
	Peri-urban	127	38.3
	Urban	43	13.0
Age of donkey (years)	<6	38	11.4
	6–10	193	58.1
	11–15	62	18.7
	16–20	23	6.9
	>20	16	4.8
Donkey sex	Male	181	54.5
	Female	150	45.2
	Gelded	1	0.3
Donkey breed	Sperki	26	7.8
	Shinghari	49	14.8
	Indian	9	2.7
	Mixed Breed	248	74.7

### Mounted Loads and %BWR

**Figure 3:**
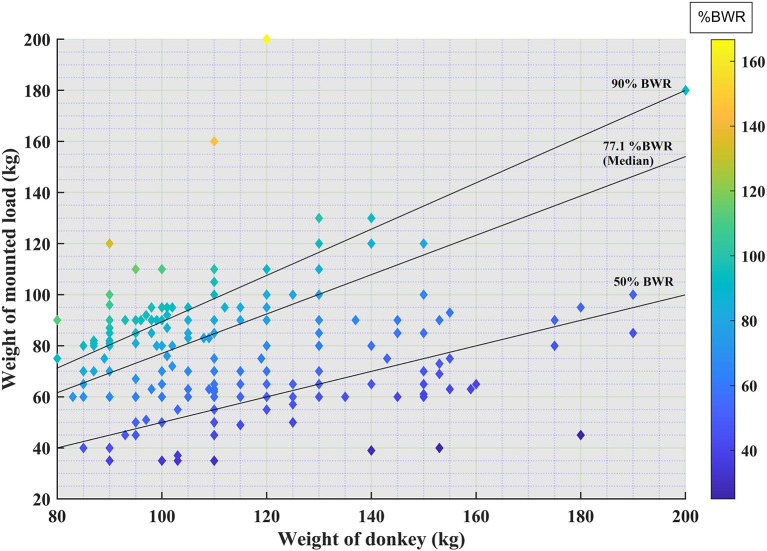
Donkey weight plotted against load carried. Lines represent 50% bodyweight ratio (BWR) carried, the median %BWR and high BWR (the upper quartile for %BWR). Lighter colors represent a higher %BWR.

The median weight for donkeys was 110 kg (IQR 100–120 kg) and the median mounted load for one trip was 81.5 kg (IQR 63–99 kg) (Figure 3). The median %BWR was 77.10% (IQR 54.50–90.70%). Overall, 87.4% donkeys carried loads above 50% BWR. Twenty-five percent of donkeys carried loads above 90 %BWR (high %BWR).

### Donkey Owners and Load Carrying

Owners reported 44.0% (*n* = 146) of donkeys were used for carrying construction-related material, 38.3% (*n* = 127) were used for carrying agricultural-related material and 17.7% (*n* = 59) were used for domestic goods. We found that 37.7% (*n* = 125) of donkeys were working on flat terrain, 5.4% (*n* = 18) on steep terrain, and 56.9% (*n* = 189) on combined flat and steep terrain. Most donkeys (*n* = 321, 96.7%) only walked during their routine daily work. In total, 41.6% (*n* = 138) of the donkey owners reported that they routinely saw lameness in their donkeys while working (Table 2).

**Table 2:** Practices of working donkey owners related to mounted load carrying.

**Variable**	**Category**	**Number**	**Percentage (%)**
Does your donkey sometimes adopt sternal recumbency after loading?	Yes	144	43.4
	No	188	56.6
What is the type of saddle you use for loading your donkey?	Wooden	162	48.8
	Cloth	31	9.3
	Plastic	4	1.2
	Hessian	133	40.1
	Don't use saddle for loading	2	0.6
Type of Load?	Construction material	146	44.0
	Agricultural load	127	38.3
	Domestic use	59	17.8
What is the working terrain?	Flat	125	37.7
	Steep	18	5.4
	Both flat and steep	189	56.9
What is the working speed?	Walk	321	96.7
	Trot	11	3.3
What are the working hours per day?	<5	156	47.0
	5–8	154	46.4
	>8	22	6.6
Have you noticed lameness signs while working?	Yes	138	41.6
	No	194	58.4

### Logistic Regression Modeling

#### Donkeys Carrying 50 %BWR

Distance traveled (km), breed of donkey, and sternal recumbency after loading were all retained in the final model. Mixed breed donkeys were 2.57 [95% Confidence Interval (CI) 1.21–5.46] times more likely to carry loads of more than fifty percent of their bodyweight, compared to other breeds of donkeys (*P* = 0.01). Donkeys traveling over 8 km per day were 7.16 (95% CI 1.47–34.79) times more likely to carry loads of more than 50% of their bodyweight, compared to donkeys traveling up to 3 km per day (*P* = 0.01). Donkeys were 4.20 (95% CI 1.30–13.55) times more likely to adopt sternal recumbency when loaded if they were loaded with more than 50% of their bodyweight, compared with donkeys loaded with less weight (*P* = 0.01) (Table 3).

**Table 3:** Multivariable regression model for 50% body weight ratio (%BWR) of load carrying donkeys.

**Variable**	**Level**	**Donkey carrying below 50 %BWR**	**Donkey carrying above 50 %BWR**	**Odds ratio (OR)**	**95% CI Lower**	**95% CI upper**	**Wald *P-*value**	**Likelihood Ratio *P-*value**
**Distance covered per day (Km)**	<4	24	89	1				<0.001
	4–8	16	38	0.50	0.23	1.08	0.08	
	>8	2	163	7.16	1.47	34.79	0.01	
**Donkey breed**	Other breeds	24	60	1				0.01
	Mixed breed	18	230	2.57	1.21	5.46	0.01	
**Does your donkey sometimes adopt sternal recumbency after loading?**	No	38	150	1				0.008
	Yes	4	140	4.20	1.30	13.55	0.01	

#### Donkeys Carrying Median %BWR

Area, age of donkey, type of load, earnings per day (PKR), and distance traveled (km) were all retained in the final model. The odds of carrying a load of more than the median %BWR in the sampled population of donkeys was higher if the donkey was working in a peri-urban [OR 2.78; 95% CI (1.16–6.63)] or urban area [12.82 OR; 95% CI (3.68–44.71)], compared to a rural area (*P* = < 0.001). Younger donkeys aged between 1 and 5 years carried more than median weight compared with donkeys aged 15 or older [11.38 OR; 95% CI (1.10–117.20); *P* = 0.03]. Donkeys carrying construction materials were more likely to carry over the median BWR [OR 5.41; 95% CI (1.69–17.26); *P* = 0.004], (Table 4).

**Table 4:** Multivariable regression model for the median percent body weight ratio (%BWR) of load carrying donkeys.

**Variable**	**Level**	**Donkey carrying below the median**	**Donkey carrying above the median**	**Odds ratio (OR)**	**95% CI Lower**	**95% CI upper**	**Wald *P-*value**	**Likelihood ratio *P-*value**
**Area**	Peri-urban	22	104	2.78	1.16	6.63	0.02	<0.001
	Rural	135	28	1				
	Urban	9	34	12.82	3.68	44.71	6.26	
**Donkey age (years)**	1–5	11	27	11.38	1.10	117.20	0.04	0.03
	6–10	67	126	5.25	0.61	45.12	0.13	
	11–15	47	12	1.82	0.18	18.08	0.61	
	>15	41	1	1				
**Type of load?**	Domestic	51	8	1				<0.001
	Agriculture	91	36	3.03	0.88	10.34	0.08	
	Construction	24	122	5.41	1.69	17.26	0.004	
**Earnings per day (PKR)**	<700	55	111	1				0.005
	700–900	54	39	0.42	0.17	1.06	0.06	
	>900	59	14	0.18	0.06	0.53	0.002	
**Distance covered per day (Km)**	<4	102	11	1				<0.001
	4–8	43	11	2.15	0.70	6.57	0.18	
	>8	21	144	14.81	4.99	43.95	1.18	

#### Donkeys Carrying High %BWR (90% BWR)

Area, age of donkey, breed, working terrain, and working hours per day were all retained in the final model. The odds of carrying a load of more than 90% of bodyweight was higher if the donkey was working in a peri-urban [OR 14.51; 95% (CI) (4.10–51.37)] or urban area [OR 8.38; 95% CI (2.15–32.67)], compared to rural areas (*P* ≤ 0.001). Mixed breed donkeys were 17.92 (95% CI 2.40–133.87) times more likely to carry loads of more than 90 percent of their bodyweight, compared to other breeds of donkeys (*P* = 0.005). Donkeys working for more than 8 h a day were 26.31 (95% CI 4.11–168.52) times more likely to carry a load more than the 90% BWR cut-off, compared to donkeys working <5 h per day (Table 5).

**Table 5:** Multivariable regression model for the high percent body weight ratio (%BWR) of load carrying donkeys.

**Variable**	**Level**	**Donkey carrying below high %BWR**	**Donkey carrying above high %BWR**	**Odds ratio (OR)**	**95% CI lower**	**95% CI upper**	**Wald *P-*value**	**Likelihood ratio *P-*value**
**Area**	Peri-urban	62	64	14.51	4.10	51.37	<0.001	<0.001
	Rural	159	4	1				
	Urban	27	16	8.39	2.15	32.67	0.002	
**Donkey age (years)**	1 to 10	149	82	28.72	4.82	171.01	<0.001	<0.001
	> 10	99	2	1				
**Donkey breed**	Other breeds	79	5	1				<0.001
	Mixed breed	169	79	17.92	2.40	133.87	0.005	
**Working terrain**	Mixed	151	38	1				<0.001
	Plains	83	42	0.31	0.14	0.67	0.003	
	Steep	14	4	223.54	14.56	3,431.07	<0.001	
**Working hours per day**	<5	151	5	1				<0.001
	5 to 8	86	68	11.95	2.48	57.55	0.002	
	>8	11	11	26.32	4.11	168.52	0.001	

## Discussion

Studying load carrying in working donkeys is important because high workload and unsafe practices contribute to poor working donkey welfare as it leads to gait disruption, ataxia, the development of abnormal behaviors, lameness, and soft tissue and bone injuries (3, 7). We explored mounted load carrying by donkeys in Pakistan and the factors associated with the weight of load carried. Despite overloading being an important donkey welfare problem (7), the current report is the first to elucidate factors associated with mounted loads carried under field conditions. One quarter of donkeys carried loads equal to 90% or more of their own bodyweight, with some donkeys estimated by their owners to be carrying more than 150% of their bodyweight. Factors including the type of load carried, the breed and age of the donkeys, and their location of work were associated with how much load the donkey carried. The variables (type of load, age of donkey, daily working hours, distance traveled and area of work) associated with donkey loading in this population have previously been associated with the poor welfare of working donkeys more broadly (7, 17, 18, 26, 33–35).

Overloading based on current recommendations (50% BWR) (23) was common, with the majority (87.4%) of donkeys reported to carry more than 50% BWR. The weight of donkeys in our study was comparable to a previous report of draft donkeys in Pakistan (36). The weight of mounted loads found in our research is also similar to previous investigations from Ethiopia (17) and India (37). However, it is suggested that donkeys can carry more than one third of their bodyweight (i.e., 40–80 kg) (38). Further, experimental research has suggested that donkeys can travel further, for longer and with less physiological impact if they are loaded with 40–50% of their bodyweight as compared to 66% of their body weight (23). Moreover, guidelines for donkeys working on beaches in the United Kingdom mandate carrying not more than 28% of bodyweight (24), however, these guidelines are not based on experimental evidence. As compared to donkeys, maximum permissible load-carrying limits suggested (based on experimental research) for native Japanese horses is 29% (39, 40), for Yonagunai ponies is 33% (41), and for Taishuh ponies is 43% of their bodyweight (40). Anecdotally, it is said that a rider should not weigh more than 10% of the horse's bodyweight in the UK, but in the US, this limit is doubled to 20% of the horse's weight; however, these guidelines are traditional rather than research-based, often impractical, and are seldom adhered to (24).

In urban areas, donkey owners were more likely to load their donkeys to more than the median and more than 90% of their bodyweight compared to rural areas. The area a working donkey lives in is a known factor for poor working equine welfare (10, 18, 26), as rural donkeys usually had fewer lesions on their body than urban donkeys and a larger proportion of urban donkeys showed moderate to severe gait deviation (i.e., lameness) than rural donkeys (10, 42). Moreover, rural donkeys work less than those in urban areas (5). Unfortunately, these authors did not define “work less” in terms of a lighter loaded weight, shorter working hours or distance traveled. However, this finding may be why fewer welfare concerns were raised for rural donkeys (10). Furthermore, donkeys in rural and urban settings have different roles within these communities and face different welfare challenges (5, 10). Due to the differences in practices between urban and rural areas, determining the welfare and socio-economic value of working donkeys in different parts of the same territory is crucial.

The type of load carried (construction, agricultural, or domestic), was associated with the weight of mounted load. Donkeys working for the transportation of construction-associated load carry more weight than donkeys carrying agricultural loads. There is currently no research comparing the type of load carried and the weight of that load. However, working donkeys that transport different types of loads experience different impacts on their health and welfare (10, 26, 33, 34, 43–46). For example, donkeys used in brick transport are 2.5 times more likely to have moderate to deep skin lesions and 3.4 times more likely to have sole surface abnormalities than those used for other purposes (26). We hypothesis that because brick is a dense material, more bricks will fit on the back of a donkey than other materials, leading to heavier loads being carried when compared to less dense agricultural or domestic loads.

Most donkeys worked for <8 h and covered a median distance of 8 km (ranges, 1–30 km) per day. The daily working hours of donkeys varies in Ethiopia (29), Mexico (33), Egypt (34), and Nepal (46). In our study, donkeys working for a greater number of hours and covering more distance per day carried more weight. This is associated with the type of workload; donkeys working for the transportation of construction associated load usually carry more weight, work for longer hours, and cover more distance than donkeys working for domestic or agricultural work, as they typically carry less, for a shorter period of time and over a shorter distance. Donkeys transporting agricultural load work less than donkeys involved with other types of work (5, 26). Moreover, donkeys work for up to 12 h a day in Ethiopia (17), and cover a distance of more than 30 km a day in Morocco (18). As the duration of work and distance traveled increases, it compromises donkey welfare (17, 18, 34). Longer working hours and increased distance covered in addition to high mounted loads are likely to lead to fatigue, and fatigue from overworking and overloading can compromise donkey welfare and productivity (34), as fatigue, heat stress, and dehydration disturb body processes and can result in organ damage and even death (47, 48). Dehydration prevents thermal conductance from the core to the periphery, increasing the risk of hyperthermia (49). Hyperlactatemia and hypercapnia induce cardiac arrhythmias during work, which can result in cardiovascular morbidity and mortality. Reduced vascular integrity caused by hypovolemia can result in peripheral edema, pulmonary edema, laminitis, and intravascular clotting (48).

Donkey age was associated with load carried in all models when donkeys were carrying more than 50% of their bodyweight. Younger animals between 1 and 5 years of age carried more load compared to older animals. In the UK it is recommended that donkeys be at least 4 years old before starting work (20). Donkeys may appear mature at the age of two, but they are not skeletally mature until they are 3–4 years old, and it has been suggested that donkeys should not carry weight or work until they are 5–6 years old to avoid osteoarthritic changes due to overworking (50, 51). Previous research has found gait abnormalities, hoof abnormalities, tendon, joint swelling, and other load-associated injuries are more prevalent in older working donkeys (17, 26, 29); we suggest that this is because they have been carrying higher levels of mounted loads throughout their young lives, and they face multiple complex issues in their older age.

In our survey, 42% of donkey owners reported seeing lameness while their donkey was working. However, a more in-depth lameness examination by a veterinarian or other appropriately trained professional would be needed to confirm this. In comparison, visual signs of lameness were observed in 15% of working equids by experts in Mexico (33), while gait abnormalities in working equids reported by experts in a wide range of countries range from 17.1 to 99.2% (26). A recent study of working donkeys pulling carts in the Faisalabad region of Pakistan found that 96% of donkeys were lame when examined by a veterinarian, despite examination being conducted while the donkey was still in harness (6). Owners in our survey reported less lameness as compared to previous reports from Pakistan; this could be due to differences in areas within Pakistan or may be due to donkey owner abilities to identify lameness. There is an assumption that owners report less lameness as compared to veterinarians and this assumption is based on surveys of horses, which have repeatedly demonstrated that owners report a lower prevalence of lameness and gait asymmetry than experts (52, 53). However, donkey owners have suggested work overload as a potential cause for lameness in Ethiopia (54) and Pakistan (6), and mule owners also recognize this issue (29). Lameness is one of the main welfare issues reported in working equids globally (6, 7, 10, 26, 29) and this is an area for important future targeted owner education. Donkeys are commonly presented with severe lameness due to their stoic demeanor, which can contribute to disease identification being delayed (55, 56). Clinical signs associated with lameness in donkeys in Pakistan includes tendinitis, joint swellings, reduced range of motion, pain on palpation, poor conformation, hoof abnormalities and back pain (36). In addition to poor welfare resulting from pain, lame horses expend more energy compared to sound horses when moving at a consistent speed. In case of animals working several hours per day, lameness may increase the demand on energy reserves in animals that already have low body condition scores (57). Alternatively, in order to compensate for lameness, donkeys may work more slowly, resulting in lower productivity and decreased earnings for impoverished owners (58).

Donkey owners reported that donkeys carrying more than 50% of their bodyweight were more likely to adopt sternal recumbency after loading irrespective to type of load and area of work. However, the adoption of sternal recumbency requires further investigation in experimental research to determine its validity as a load quantifying factor in working donkeys. This study's two major limitations are convenience sampling and owner-reported weights, both of which are unavoidable in this context. Because the data are based on answers gathered during owner interviews, the accuracy of the data must be carefully considered (18). Furthermore, the reliability of ‘owner information' has not been validated and may be imperfect or biased due to owner reporting of perceived “correct” answers (18).

Factors associated with mounted load carrying in working donkeys in Pakistan have been identified in this study. At present, there are no evidence-based guidelines for load carrying limits in working donkeys (7), although guidelines for pulled load exist in some countries (8). This survey is a starting point for the development of evidence-based recommendations for donkey loading. Quantified loading thresholds or predictors of overloading can then be used by non-governmental organizations (NGOs), legislators, and other decision-makers working with working donkeys to restrict overloading and optimize donkey welfare.

It is clear based on the use and role of the donkeys in this study, it is likely that recommending that they are only loaded at 50% of bodyweight will not be feasible. We need a greater understanding regarding the motivations and perceptions of owners around donkey loading, and the socio-economic role that load carrying donkeys play for their owners. While the welfare of the donkey is important, and a consideration here, donkey welfare can only be improved alongside community recognition of the issue, and a general improvement of human living conditions.

## Conclusion

Our research has provided valuable information on the demographics of working donkeys, and the factors associated with mounted load carried by working donkeys in Pakistan. Factors including type of load carried, the breed, sternal recumbency, and age of the donkeys, and their location of work were associated with how much load the donkey carried. Overloading based on current recommendations (50% BWR) was common, and 87.4% of donkeys were carrying more than 50% of their bodyweight in the survey region. As overloading is one of the most common welfare issues in working donkeys, this is an area in which future education efforts should be targeted.

## Data Availability Statement

Due to data privacy concerns and conditions of the verbal consent given by the owners, the full dataset is not publicaly available. Queries can be made to the corresponding author.

## Ethics Statement

The studies involving human participants were reviewed and approved by Human Subjects Ethics Sub-Committee, City University of Hong Kong (Approval Reference No. JCC2021AY003). Written informed consent for participation was not required for this study in accordance with the national legislation and the institutional requirements.

## Author Contributions

All authors were involved in the preparation of the manuscript, gave final approval of this manuscript, read, and agreed to the published version of the manuscript.

## Funding

This project was funded by City University of Hong Kong (Grant Number 9610463).

## Conflict of Interest

SR is an independent epidemiological consultant who consults under Equine Veterinary Consultants (EVC) Limited. The remaining authors declare that the research was conducted in the absence of any commercial or financial relationships that could be construed as a potential conflict of interest.

## Publisher's Note

All claims expressed in this article are solely those of the authors and do not necessarily represent those of their affiliated organizations, or those of the publisher, the editors and the reviewers. Any product that may be evaluated in this article, or claim that may be made by its manufacturer, is not guaranteed or endorsed by the publisher.
