# Supplementary material for: Welfare Concerns for Mounted Load Carrying by Working Donkeys in Pakistan
Source: Front Vet Sci. 2022 May 27;9:886020. doi: 10.3389/fvets.2022.886020 (PMC9186103; doi:10.3389/fvets.2022.886020)
Supplement: Supplementary file 2 [file Table_2.DOCX]

1


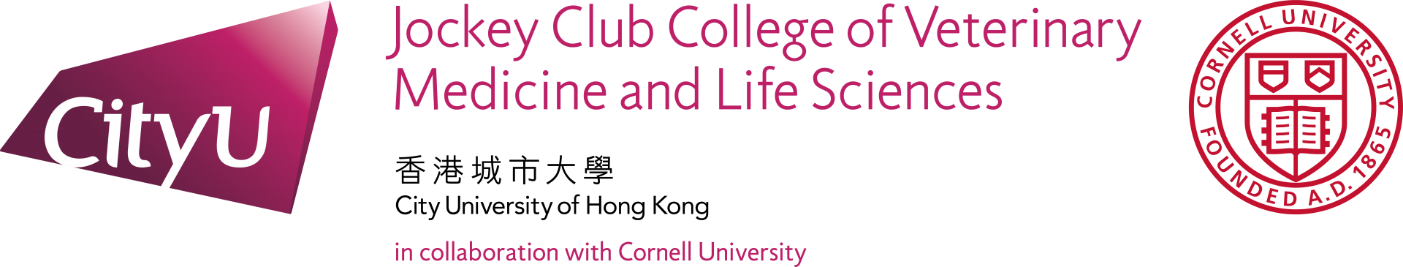


**Demographics of the owner, and signalment of the donkey**

- **Owner age (years):**

**(a)** less than 31 **(b)** 31-40 **(c)** 41-50 **(d)** More than 50

- **Owner Gender:**

**(a)** Male **(b)** Female

- **Region:**

**(a)** Swat **(b)** Attock **(c)** Faisalabad **(d)** Cholistan

- **Area:**

**(a)** Rural **(b)** Peri-Urban **(c)** Urban

- **Age of donkey (years):**

**(a)** less than 6 **(b)** 6-10 **(c)** 11-15 **(d)** 16-20 **(e)** More than 20

- **Sex of donkey:**

**(a)** Male **(b)** Female **(c)** Gelded

- **Breed of donkey:**

**(a)** Sperki **(b)** Shinghari **(c)** Indian **(d)** Mix breed

# Loading practices

- **Weight of donkey (kg):** ________________
- **How much load you put on one donkey for one trip/visit? (kg)** ________________
- **Does your donkey sometimes adopt sternal recumbency after loading?**

**(a)** Yes **(b)** No

- **What is the type of saddle you use for loading your donkey?**

**(a)**Wooden (**b)** Cloth **(c)** Plastic **(d)** Bhoori (Hessian)

**(e)** Don’t use saddle for loading

- **Type of Load?**

**(a)** Construction material **(b)** Agricultural load **(c)** Domestic use

- **How much distance does your donkey usually cover per day while under load (in km)?** __________________
- **What is the working terrain?**

**(a)** Flat **(b)** Steep **(c)** Both flat and steep

- **What is the working speed?**

**(a)** Walk **(b)** Trot

- **What are the working hours per day?**

**(a)** less than 5 **(b)** 5-8 **(c)** more than 8

- **Have you noticed lameness signs while working?**

**(a)** Yes **(b)** No

- **What is the donkey earning per day? (PKR)**
